# Efficacy and Safety of a Topical Formulation Containing Trihydroxybenzoic Acid Glucoside and α‐Arbutin, Applied Along With a Sunscreen: A Noncomparative, Prospective, Interventional Study in Indian Females With Facial Melasma or Dark Spots

**DOI:** 10.1111/jocd.70017

**Published:** 2025-02-12

**Authors:** Mukesh Gabhane, Raji Patil, Shruti Dharmadhikari, Priyank Shah, Chintan Khandhedia, Suyog Mehta

**Affiliations:** ^1^ Ex‐Employee, Medical Affairs Sun Pharma Laboratories Limited Mumbai India; ^2^ Dermatologist Mascot Spincontrol India Pvt. Ltd. Mumbai India; ^3^ Medical Affairs Sun Pharma Laboratories Limited Mumbai India

**Keywords:** α‐arbutin, dark spots, hyperpigmentation, melanin content, melasma, trihydroxybenzoic acid glucoside

## Abstract

**Background:**

Skin hyperpigmentation is a dermatological concern for pigmented skin phototypes. Despite availability of multiple treatment options, hyperpigmentation management continues to be a challenge.

**Aims:**

Efficacy and safety assessment of the skincare regimen [topical formulation of trihydroxybenzoic acid glucoside 10% (THBG) and α‐arbutin 2% (twice‐daily) + sunscreen (once daily)] in Indian females with pigmentary spots (facial dark spots or melasma).

**Methods:**

This prospective, open‐label, single‐arm, interventional study was conducted in Indian females (*n* = 124), aged 18–45 years, having FitzPatrick skin type III–IV, dull skin, and facial dark spots or melasma. Efficacy of 90‐day skincare regimen was evaluated using mexameter, modified melasma area and severity index (mMASI) score, cross‐polarized light photography, chromameter, and color–luminosity–brightness–transparency (CLBT) technique. Student's *t*‐test (paired data) was used for statistical analysis.

**Results:**

In efficacy analysis [modified intention‐to‐treat (mITT) population], 120 subjects were included. Melanin content of pigmentary spots (on mexametry) significantly reduced (−16.3%, *p* < 0.001) at Day 90 versus baseline. Significant reduction in severity of melasma was observed (−18.4% in mMASI score) at Day 90. Significant improvement in ΔEab (−8.7%) (change in color difference between pigmentary spot and normal skin) on cross‐polarized photographs, in skin‐brightening parameters L* (relative brightness/lightness) (−2.7%), ITA° (individual typology angle) (−37.7%), and ΔE* (skin‐tone evenness) (−19.0%) using chromameter. Significant improvement in skin radiance was observed in CLBT parameters, color (pink: 32.8%, yellow: –5.5%, and olive: −7.8%), luminosity (6.0%), brightness (4.6%), and transparency (0.2%). Regimen did not cause itching, burning, or irritation.

**Conclusions:**

Skincare regime of 10% THBG and 2% α‐arbutin along with sunscreen was effective in reducing pigmentary spots (dark spots and melasma) and was well tolerated in Indian women.

**Trial Registration:** Clinical Trial Registry of India (CTRI Reg. No. CTRI/2021/11/038345; date: 30/11/2021)

## 1 Introduction

Skin hyperpigmentation represents one of the major dermatological concerns for populations with pigmented skin phototypes. Over the years, pigmentation of human skin has evolved depending on genetic, climatic, and cultural factors [1]. Asians, especially Indian skin, have greater susceptibility to develop pigmentary disorders of the skin due to their skin type than other demographic populations [2]. Hyperpigmentary conditions including melasma, postinflammatory hyperpigmentation, periorbital hyperpigmentation, and solar lentigines are frequent in Indian population [2]. A study from Western India reported 10.8% prevalence of pigmentary disorders [3]. Hourblin et al. [4] recorded heterogeneity in skin tones in more than 80% population across four cities of India; melasma, hyperpigmented spots, pigmented macules, and dark circles were responsible for this skin color diversity. Hyperpigmentation or dark spots are age dependent and commonly occur on face, extensor forearms, and trunk as a consequence of photodamage [2]. Dark spots start to appear in women around the age of 30 years [4]. Melasma has a higher incidence rate in India (0.25%) than other pigmentary disorders [3], and is more prevalent in Indian females than males (4:1 ratio), aged between 14 and 54 years [5]. Melasma is reported to occur in 30% middle‐to‐older (41–65 years) women population [4]. Melasma, an acquired condition of hyperpigmentation, typically appears on the face in centrofacial (cheeks, forehead, upper lip, and nose), malar (cheeks and nose), or mandibular (mandibular area of cheeks) pattern [6, 7]. A multicentric cross‐sectional study involving four (east, west, north, and south) regions of India found onset of melasma on the cheeks in 65.2% of subjects, followed by nose (27%), forehead (22.8%), upper lip (8.4%), and chin (8.4%) [8]. Genetic predisposition, thyroid dysfunction, sun exposure, pregnancy, use of cosmetics and oral contraceptives, and exposure to fire/heat during cooking are considered as the attributable risk factors for melasma [6, 8]. Untreated facial hyperpigmentary conditions greatly contribute to psychosocial distress and affect the individual's quality of life [9].

Localized hyperpigmentation, like melasma can be treated with topical retinoids, azelaic acid, hydroquinone, chemical peels, or cosmeceuticals, and using sunscreen and physical barriers to get protection from the sun [10]. Hydroquinone has been extensively prescribed for hyperpigmentation and melasma treatment, but chronic use is associated with safety concerns [11]. In an effort to provide an alternative to hydroquinone, arbutin, a compound of hydroquinone has been studied for 30 years [11], and found to be effective in reducing area and severity index of melasma [6, 12] with lesser side effects [13]. Arbutin enhances antioxidant capacity of cells by reducing reactive oxygen species (ROS) through nuclear factor erythroid 2‐related factor 2 antioxidant responsive elements (Nrf2‐ARE) pathway, which in turn inhibits tyrosinase‐mediated melanin synthesis [11]. Trihydroxybenzoic acid glucoside (THBG) with photoprotectant properties is used for protection of the skin against ultraviolet (UV) rays and for improving skin brightness, redness, yellowness, and brownness by decreasing skin melanin content [14]. In addition, THBG inhibits ROS production and prevents UV‐induced DNA damage. It also controls nuclear factor kappa B (Nf‐κB) pathway and expression of microphthalmia‐associated transcription factor, involved in melanogenesis process [15].

A novel formulation, Bristaa Intense Cream containing 10% THBG and 2% α‐arbutin has been developed by an Indian multinational pharmaceutical company. A previous prospective, single‐arm study had evaluated efficacy and safety of same formulation along with a sunscreen for 56 days in 36 Indian females with facial dark spots or melasma. Although the study established that the topical formulation was well tolerated and efficacious in hyperpigmentation management, the short study duration and a small sample size were the limitations [15]. Therefore, the current study was designed to evaluate the efficacy, safety, and cosmetic appeal of skincare regimen with combination of Bristaa Intense Cream and Photostable Gold Sunscreen Gel in 124 female subjects with pigmentary spots (dark spots or melasma) after 90 days of application.

## 2 Materials and Methods

### 2.1 Study Design

This was a noncomparative, nonblinded, prospective, interventional study conducted between December 2021 and June 2022. The objective of the study was to assess the efficacy, safety, and cosmetic appeal of skincare regimen of Bristaa Intense Cream along with Photostable Gold Sunscreen Gel in female subjects with dark spots or melasma.

### 2.2 Study Participants

Indian female subjects aged 18 and 45 years with dull skin (i.e., skin that lacks brightness and has uneven tone based on visual assessment of the experts), Fitzpatrick skin type III–IV, and having dark spots (with at least one dark spot of ≥ 3.5 mm in diameter) or visible melasma on face with modified melasma area and severity index (mMASI) score between 1 and 8 were included in the study. Written informed consent was obtained from all participants.

Females with chronic dermatosis, diabetes, progressive asthma, epilepsy, thyroid disorders, cutaneous hypersensitivity, allergy to cosmetic or food products or latex, or suntanned skin on the studied areas were excluded. Moreover, females who received aspirin‐based products, anti‐inflammatories, antihistamines, corticosteroid therapy treatment for > 1 year, hormonal contraception or hormone replacement therapy in last 3 months, general anesthesia for > 1 h in last 6 months, or other treatment that could interfere with the evaluations of the study were excluded. In addition, smokers, pregnant or breastfeeding females, or who received medicinal treatment responsible for hyperpigmentation or whitening of the skin, or changed cosmetic habits 14 days prior to the study, or who have undergone beauty treatments or physical and/or chemical treatments of the spots were excluded. Females, who were participating in another study, had suntanned skin/cosmetic product/make‐up applied on the screening day, or applied beauty treatment products (skin cleansing/exfoliation/scrub/mask/self‐tanning products) or products with anti‐wrinkle action or with depigmenting action, were also excluded.

### 2.3 Ethical Approval and Study Site

The protocol and study documents were reviewed and approved by the Institutional Ethics committee named “ETHOS.” The study was registered in Clinical Trial Registry of India (CTRI Reg. No. CTRI/2021/11/038345; Date: 30/11/2021), and conducted as per ICH GCP and all the applicable regulatory requirements. The study was performed at a global clinical research organization located in India.

### 2.4 Intervention and Study Procedure

The skincare regimen used in this study, Bristaa Intense Cream, a skin‐brightening cream and Photostable Gold matte finish, and a sunscreen gel with sun protection factor (SPF) of 55 were provided by an Indian multinational pharmaceutical company. Bristaa Intense Cream (20 g) (Batch No. SGV0020) containing Brightenyl or THBG (10%), α‐arbutin (2%), Flashwhite unispheres (1%), and Unitamuron H‐22 (1%) was applied twice daily on the whole face. Photostable Gold Sunscreen Gel (20 g) (Batch No. SXC1371A) consisting of Uvinul MC80 (7.50%), Tinosorb M (5%), Tinosorb S (2.80%), Uvinul T150 (3%), and Uvinul A plus (2.50%) was applied once daily in the morning on the whole face. The same daily regimen was followed by the subjects for study duration of 90 days. During every visit, a period of 20 min for acclimatization was provided to every subject in an air‐conditioned room (temperature: 20°C–25°C and relative humidity: 50% ± 10%) before any study procedures and assessments were carried out. Baseline measurements were done on Day 0. Subjects visited the study site for follow‐up on Days 28, 42, 56, 70, and 90. They were instructed not to apply the test products on the morning of follow‐up visits.

### 2.5 Study Endpoints

#### 2.5.1 Primary Endpoint

The primary endpoint was percent reduction in melanin content of pigmentary spots (dark spots or melasma) assessed through mexametry at Day 90 compared to baseline.

##### 2.5.1.1 Mexametry: Melanin Content

The melanin contents were evaluated using a Mexameter in order to calculate and compare erythema and melanin [16]. Emission of three specific wavelength lights by the Mexameter MX 18 (Courage and Khazaka, Germany) probe and their reflection by the skin were measured to calculate light absorbed by the skin. At baseline, the location of pigmentary spot was determined by a cutaneous marking on the instrumental measurement sites by calculating the distance of the spot from the nose and eyes. Three measurements were taken for each subject at each examination time point. Results obtained for melanin were expressed in arbitrary units. Mean of the three measurements was recorded. Decreased mexametry readings with application of the product are an indicator of reduced melanin content of the pigmentary spots (dark spots and melasma).

#### 2.5.2 Secondary Endpoints

Secondary endpoints included assessment of percent reduction in melanin content of pigmentary spots assessed through mexametry on Days 28, 42, 56, and 70 (dark spots or melasma). Other secondary endpoints included percent improvement in mMASI score; percent improvement in skin brightening (L* and individual typological angle or ITA° values) and even skin tone (ΔE* value) through chromametry; percent improvement in skin radiance in terms of reduction in olive and yellow color, and improvement in pink color, luminosity (*L*) score, brightness (B) score, and transparency (T) score through color–luminosity–brightness–transparency (CLBT) methodology; adverse events; and percent panel agreement based on subject's self‐evaluation questionnaire score for efficacy, safety, and cosmetic appeal of the skincare regimen along with cosmetic appeal of sunscreen gel. All of these secondary endpoints were assessed at Days 28, 42, 56, 70, and 90. Percent reduction in ΔEab value, number, and area of pigmentary spots (dark spots or melasma) through cross‐polarized photography was assessed on Days 56, 70, and 90. The same dermatologist and technician performed all the evaluations.

##### 2.5.2.1 mMASI Score: Severity Index of Melasma

**FIGURE 1:**
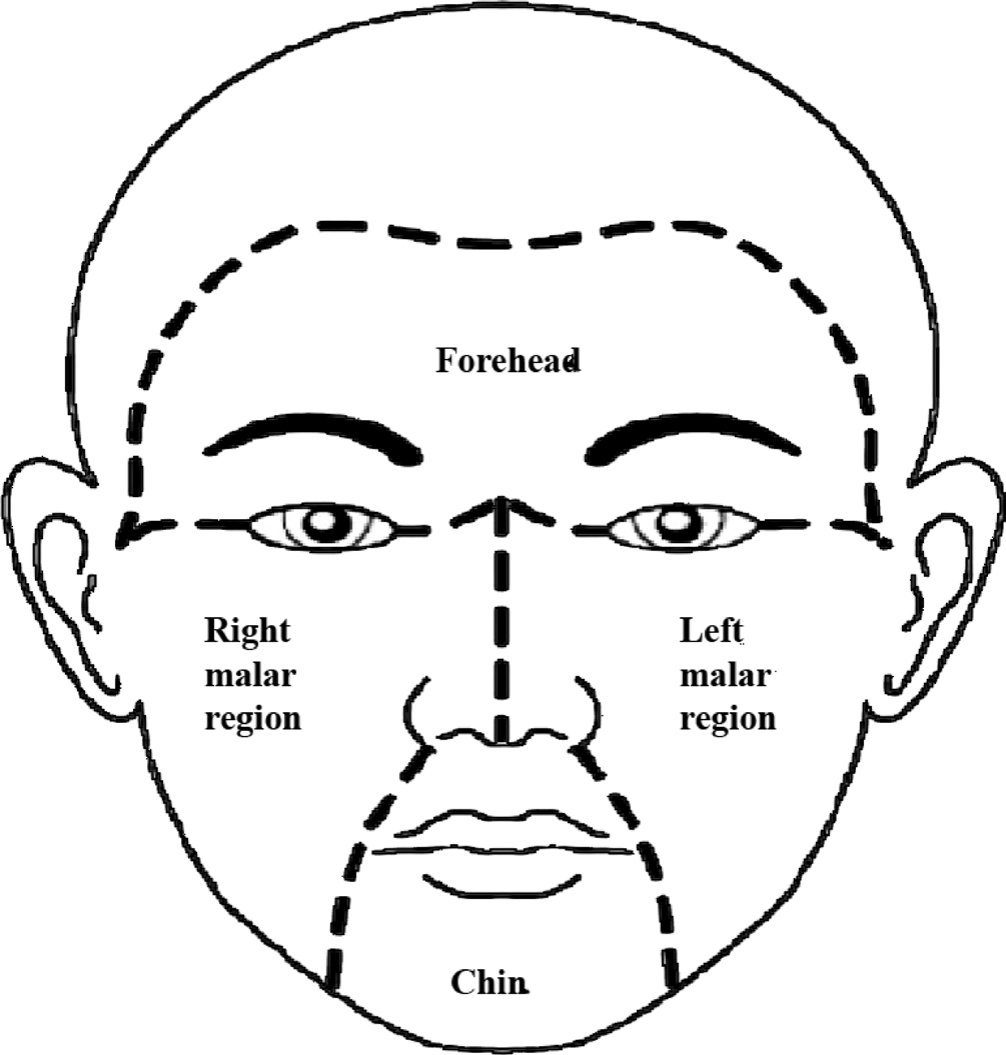
Melasma area of involvement.

The mMASI score is widely used to calculate melasma severity [17]. The dermatologist assessed the severity of melasma using mMASI score at Days 28, 42, 56, 70, and 90 in subjects, who had melasma, and who completed the study. Subjective assessment of two factors was carried out, namely, area of involvement and darkness on the forehead, right malar region, left malar region, and chin (Figure 1), corresponding to 30%, 30%, 30%, and 10% of the total face, respectively. Area of involvement in each of these four areas was given a numeric value of 0 to 6 (0: absent; 1: < 10%; 2: 10%–29%; 3: 30%–49%; 4: 50%–69%; 5: 70%–89%; and 6: 90%–100%) and darkness as 0 to 4 (0: absent; 1: slight; 2: mild; 3: marked; and 4: severe). The range of the total score was 0 to 24. Reduction in mMASI score demonstrates efficacy of the tested product in terms of melasma. The mMASI score was calculated using the following formula:

$$\begin{array}{c} \text{mMASI score}=0:3\left(A\right)\;\left(f\right)\;D\;\left(f\right)+0:3\left(A\right)\;\left(\mathrm{lm}\right)\;D\;\left(\mathrm{lm}\right)+0:3\left(A\right)\;\left(\mathrm{rm}\right)\;D\;\left(\mathrm{rm}\right)\\ \;+0:1\left(A\right)\;\left(c\right)\;D\;\left(c\right) \end{array}$$

A: Area of involvement, D: darkness, f: forehead, rm.: right malar, lm: left malar, c: chin.

##### 2.5.2.2 Cross Polarized Photographs: Color and Morphology Analysis

Cross‐polarization photographs are helpful for the examination of pigmentation, skin texture, and vascularity. It provides structural information on superficial skin lesions and it is useful for the assessment of skin chromophores excited by UV‐A radiation [18]. Percent reduction in ΔEab value, number of pigmentary spots, and total area occupied by pigmentary spots were assessed through analysis of cross‐polarized photographs at Days 56, 70, and 90. To accomplish this, high‐resolution photographs of three‐fourth or the whole face were obtained in completely reproducible lighting conditions, in cross‐polarized light using Nikkor 60 mm lens, equipped with a filter in a dark room. To ensure good reproducibility of the acquisition condition, the subjects were instructed to wear a mobcap and a black cloth, and photographs of the face were taken after keeping the face on an optical measurement bench, Visio‐Face, developed by Spincontrol. Detection of one or several spots was done using a software developed by Spincontrol. Value of ΔEab represents the color difference between selected spot and normal skin on a scale from 0 to 100, where 0 is less color difference and 100 is complete distortion. The morphological analysis (number as well as total area of pigmentary spots in the region of interest) was carried out from a selection of several spots on each cheek.

Color analysis was carried out from a single pigmentary spot (one selected spot per cheek from either the dark spot or melasma). The studied parameter was the ∆E*ab parameter (on a scale of 0 to 100, where 0 is less color difference and 100 is complete distortion), which in this case represents the difference in the color between the spot and the normal skin through image analysis. Reduction in morphology parameters and/or in the color parameter suggested a decrease in the visibility of the pigmentary spots (dark spot or melasma).

##### 2.5.2.3 Chromametry: Skin Brightness and Skin Tone Analysis

Chromametry is used to calculate photoprotective activity and skin surface redness [19]. Improvement in skin brightening (L* and ITA° values) and even skin tone (ΔE* value) was assessed on Days 28, 42, 56, 70, and 90 using chromameter. Both cheekbones and six different sites on face were selected for measurement of skin brightening and skin tone evenness, respectively. The locations were determined by a cutaneous marking on the instrumental measurement sites. Three measurements (L*, a*, and b*) were carried out on the study areas for each subject at each examination time:

- L* indicates the relative brightness on a scale from 0 to 100, where 0 is total black and 100 is total white.
- a* describes colors ranging from green (negative value) to red (positive value).
- b* indicates colors ranging from blue (negative value) to yellow (positive value).

$$\mathrm{ITA}{}^{\circ}=\text{Arctan}\;\left[\left(L^{*}50\right)/b^{*}\right]\times \left(180/\pi \right)$$

ΔE* = [(L*si − L*sj)^2^ + (a*si − a*sj)^2^ + (b*si − b*sj)^2^]^1/2^, where si and sj means two different sites defined on the face, and ΔE* represents skin tone and measures color difference between two sites on a scale from 0 to 100, where 0 is less color difference and 100 indicates complete distortion. Decreased ΔE parameter corresponds to an improvement of the even skin tone (skin homogeneity), and increased L* and ITA° value indicates skin brightening.

##### 2.5.2.4 CLBT Methodology: Skin Radiance

The CLBT methodology assesses different descriptors of the skin tone: coloring, brightness, luminosity, and transparency of facial skin [20]. Improvement in skin radiance in terms of elevated pink color and reduced olive and yellow color (*C*) complexion score, and elevated luminosity (*L*), brightness (*B*), and transparency (*T*) scores were evaluated on Days 28, 42, 56, 70, and 90 using CLBT technique. The measurements were performed on whole face. Colors of facial skin (yellow/pink/olive) are graded based on its saturation (from 10% to 100%). Increase in pink color saturation gives “looks healthier” effect, and decrease in yellow and olive color saturation gives “looks good” and “looks good and healthier” effect, respectively. Luminosity is intensity of light, reflected on the salient areas of the face; brightness is uniformity of the skin color; and transparency is related to thickness of the skin. Luminosity, brightness, and transparency are graded based on analogical scale of 0–10.

##### 2.5.2.5 Self‐Evaluation Questionnaire

The subjects were asked to answer self‐evaluation questionnaire in order to record their overall opinion and their attitude toward the efficacy, safety, and cosmetic appeal of the products under test. Subject self‐evaluation questionnaire score for efficacy (reduction in melasma/ dark spots, improvement in skin brightening, even skin tone, and skin radiance) and safety (questions regarding product‐related itching, irritation, and burning sensation to the skin) of the treatment regime was assessed on Days 28, 42, 56, 70, and 90. In addition, subject self‐evaluation questionnaire score for cosmetic appeal of sunscreen gel [nonsticky (no sticky residue on skin surface), nonoily (without shininess), feels smooth (velvety), does not look white (white residue), matte finish (not shiny), and light feel (weightless) on application] and skincare regimen for their fragrance, quick absorption, stickiness, and proper spread were noted at baseline and on Day 90, respectively. The answers were scored as 1: completely agree, 2: somewhat agree, 3: somewhat disagree, and 4: completely disagree. To evaluate the efficacy and the appreciation of the products for each item, two percentages Z1 and Z2 were calculated as follows:

$$\text{Z1}=\text{Favorable opinion}\;\left(1:\text{Completely agree}+2:\text{Somewhat agree}\right)$$

$$\text{Z2}=\text{Unfavorable opinion}\;\left(3:\text{Somewhat disagree}+4:\text{Completely disagree}\right)$$

##### 2.5.2.6 Safety Evaluation

Safety of the product was assessed by the dermatologist based on clinical signs (observed by the dermatologist) and functional signs (felt by the subjects and reported to the dermatologist) at baseline and throughout the study. Clinical signs including erythema, edema, dryness, scaling, and peeling, and functional signs including itching and tingling were scored as 0: none, 1: slight, 2: moderate, and 3: severe. Adverse event was defined as any untoward medical occurrence or symptom, sign, or significant abnormal laboratory finding (related to the treatment or not) if occurred during the study period. Serious adverse event was defined as an untoward life‐threatening medical occurrence that may result in hospitalization, persisting disability or incapacity, congenital anomalies, birth malformations, or death.

### 2.6 Sample Size

Assuming a minimum “reduction in melanin content” of 15 units with a standard deviation (SD) of 55 with two‐sided significance level of 5% and power of 80%, approximately 106 evaluable subjects were considered to be required in the study. To allow 15% dropout, a total of 124 subjects were required to be enrolled in the study.

### 2.7 Statistical Analysis

Efficacy analysis included modified intention‐to‐treat (mITT) population who used the test product at least once and recorded at least one assessment postbaseline. All enrolled subjects, who completed the study period as per approved protocol without any major protocol deviation/protocol violation, were included in per protocol (PP) population. Safety analysis was performed for all enrolled subjects who used at least one dose of the study product. Continuous variables were expressed in the form of mean ± SD. Normally distributed data were compared using Student's *t* test. SigmaStat (V3.5, California, USA) was used for all statistical calculations. Level of significance was considered as *p* < 0.05.

## 3 Results

### 3.1 Demographics and Baseline Characteristics

**FIGURE 2:**
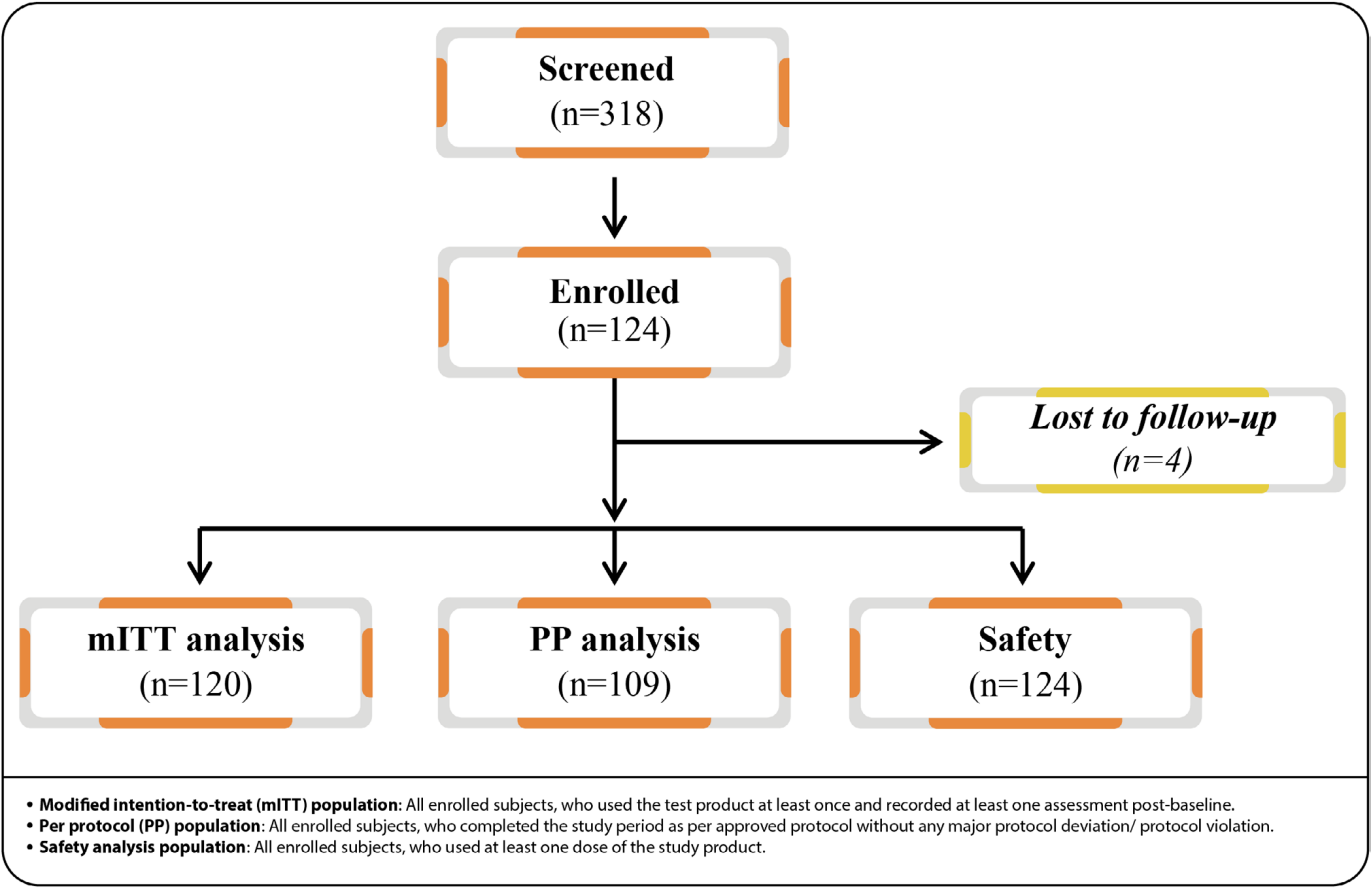
CONSORT flow of subject disposition.

A total of 318 female subjects were screened for eligibility, of which 124 fulfilled the eligibility criteria and were enrolled in the study. Efficacy analysis was performed in mITT population (*n* = 120). PP population included 109 subjects. Safety analysis included 124 subjects (safety population) (Figure 2). Mean age ± SD of all included subjects (*n* = 124) was 40.9 ± 4.7 years. At baseline, mean mMASI score ± SD (*n* = 62, subjects with melasma who were included in the study), mean number of spots ± SD (*n* = 124, who used test product at least once), and mean total area occupied (pixels^2^) by the spots ± SD (*n* = 124, who used test product at least once) were 4.8 ± 1.64, 2.59 ± 2.14, and 13935.33 ± 15215.03, respectively. Baseline demography is shown in Table 1.

**TABLE 1:** Baseline demography.

Characteristics	Value (mean ± SD)
Age (years) (*n* = 124)	40.9 ± 4.7
mMASI score (*n* = 62)	4.80 ± 1.64
Number of spots (*n* = 124)	2.56 ± 2.12
Total area occupied by spots (pixel^2^) (*n* = 124)	13635.77 ± 15057.14

### 3.2 Study Outcomes

#### 3.2.1 Primary Outcome

##### 3.2.1.1 Melanin Content (Mexametry) at Day 90

Primary endpoint was percent reduction in melanin content of pigmentary spots as measured by mexametry. Melanin content was significantly reduced by 16.3% from baseline to last follow‐up, that is, on Day 90 (439.62 ± 78.9 to 368.94 ± 67.86; *p* < 0.001, Student's *t* test [paired data] used for comparison).

#### 3.2.2 Secondary Outcomes

##### 3.2.2.1 Melanin Content (Mexametry) at Days 28, 42, 56, and 70

**FIGURE 3:**
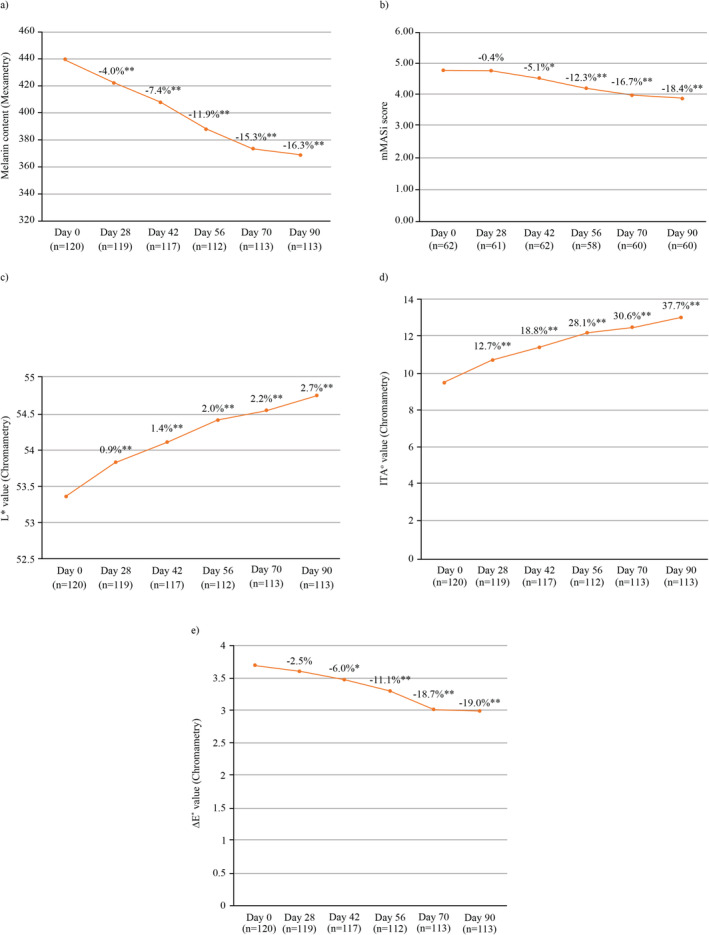
Percent change from baseline (Day 0) in (a) melanin content through mexametry, (b) mMASI score, (c) L* value, (d) ITA° value, and (e) ΔE* value using Student's *t* test (paired data). ^ns^Not significant, *p < 0.01, **p < 0.001.

Mean melanin content on Days 28, 42, 56, and 70 was 422.39 ± 78.56, 408.05 ± 76.1, 388.08 ± 70.99, and 373.42 ± 69.97, respectively. Melanin content of pigmentary spots as measured using mexametry (dark spots and melasma) was decreased significantly (*p* < 0.001, Student's *t* test for paired data used for comparison) by 4.0% as early as by Day 28, and further by 7.4% (Day 42), 11.9% (Day 56), and 15.3% (Day 70) compared to baseline (Figure 3a). Similar results were observed with reduction in melanin content of pigmentary spots in PP analysis set (Figure S1a).

##### 3.2.2.2 mMASI Score

Significant improvement in mMASI score was observed at Day 42 from baseline (4.8 ± 1.64 to 4.55 ± 1.65; reduction in score by 5.1%, *p* = 0.0020). This improvement continued for rest of the study period (Day 56: 4.24 ± 1.69, Day 70: 4.01 ± 1.61, and Day 90: 3.92 ± 1.66; *p* < 0.001 for all), as shown in Figure 3b. This reduction in mMASI score indicated the efficacy of the regimen in terms of reduction in severity of melasma. mMASI score in PP analysis set showed similar results and is presented in Figure S1b. Student's *t* test (paired data) was used to calculate statistical significance.

##### 3.2.2.3 Cross‐Polarized Photography

Analysis of cross‐polarized photographs revealed significant reduction (8.7%, *p* < 0.001) in percent value of ΔEab on Days 56, 70, and 90 compared to baseline using Student's *t* test (paired data). No significant change was observed in the number of pigmentary spots and total area occupied by the spots throughout the study (Table 2). Data on cross‐polarized photography in PP analysis set is given in Table S1.

**TABLE 2:** Evaluation of efficacy parameters in the study participants (mITT analysis set).

Endpoints	Study visit	Sample size	Mean ± SD	Average change vs. baseline	*p* vs. baseline
Cross‐polarized light photography					
ΔEab	Day 0	120	8.36 ± 2.14		
	Day 56	112	7.34 ± 1.85	−14.3%	< 0.001**
	Day 70	113	7.49 ± 1.85	−12.3%	< 0.001**
	Day 90	113	7.81 ± 1.9	−8.7%	< 0.001**
Number of spots in region of interest	Day 0	120	2.59 ± 2.14		
	Day 56	112	2.73 ± 2.52	6.2%	0.36
	Day 70	113	2.39 ± 2.29	−8.9%	0.14
	Day 90	113	2.5 ± 1.95	−5.1%	0.75
Total area occupied by spots (pixels^2^)	Day 0	120	13935.33 ± 15215.03		
	Day 56	112	14143.39 ± 14974.52	1.5%	0.36
	Day 70	113	14 193 ± 14 558	−1.1%	0.44
	Day 90	113	14834.02 ± 16289.98	3.4%	0.29
Skin radiance by CLBT methodology					
Pink color	Day 0	120	10.69 ± 6.84		
	Day 28	119	10.8 ± 6.98	0.7%	0.50
	Day 42	117	11.52 ± 7.5	6.8%	< 0.001**
	Day 56	112	12.69 ± 7.72	17.8%	< 0.001**
	Day 70	113	13.58 ± 8.11	25.8%	< 0.001**
	Day 90	113	14.34 ± 8.39	32.8%	< 0.001**
Yellow color	Day 0	120	35.57 ± 5.32		
	Day 28	119	35.34 ± 5.28	−0.4%	0.25
	Day 42	117	35.57 ± 5.08	0.1%	1.00
	Day 56	112	34.49 ± 4.7	−3.0%	< 0.001**
	Day 70	113	33.79 ± 4.24	−4.8%	< 0.001**
	Day 90	113	33.54 ± 4.12	−5.5%	< 0.001**
Olive color	Day 0	120	33.83 ± 4.19		
	Day 28	119	33.85 ± 4.21	0.0%	1.00
	Day 42	117	33.7 ± 4.07	−0.2%	0.38
	Day 56	112	32.19 ± 3.53	−4.6%	< 0.001**
	Day 70	113	31.59 ± 3.55	−6.3%	< 0.001**
	Day 90	113	31.12 ± 3.52	−7.8%	< 0.001**
Luminosity	Day 0	120	3.8 ± 0.22		
	Day 28	119	3.82 ± 0.23	0.5%	< 0.001**
	Day 42	117	3.9 ± 0.24	2.8%	< 0.001**
	Day 56	112	3.95 ± 0.24	4.3%	< 0.001**
	Day 70	113	3.99 ± 0.25	5.1%	< 0.001**
	Day 90	113	4.02 ± 0.26	6.0%	< 0.001**
Brightness	Day 0	120	3.73 ± 0.36		
	Day 28	119	3.73 ± 0.36	0.0%	0.50
	Day 42	117	3.79 ± 0.37	1.4%	< 0.001**
	Day 56	112	3.85 ± 0.38	3.3%	< 0.001**
	Day 70	113	3.88 ± 0.38	3.9%	< 0.001**
	Day 90	113	3.91 ± 0.37	4.6%	< 0.001**
Transparency	Day 0	120	3.62 ± 0.3		
	Day 28	119	3.63 ± 0.3	0.0%	1.00
	Day 42	117	3.63 ± 0.31	0.0%	0.13
	Day 56	112	3.62 ± 0.31	0.0%	0.31
	Day 70	113	3.63 ± 0.31	0.1%	0.06
	Day 90	113	3.63 ± 0.31	0.2%	< 0.001**

*Note:* Student's *t* test (paired data) used for calculation of *p* value.

** *p* < 0.001.

##### 3.2.2.4 Chromametry

The findings of chromametry assessments demonstrated significant (*p* < 0.001) percent improvements in L* and ITA° values at all posttreatment visits (Figure 3c,d). The baseline values of L* and ITA° were 53.34 ± 3.23 and 9.63 ± 9.42, respectively, that increased significantly (*p* < 0.001) to 54.74 ± 3.13 and 13.18 ± 8.52, respectively, on the final follow‐up. From baseline, significant (*p* = 0.005) improvements in even skin tone in terms of percent reduction in ΔE* value, started from posttreatment Day 42 (3.47 ± 0.89), were also recorded. In comparison to baseline, the ΔE* value was significantly (*p* < 0.001) reduced by 11.1% on Day 56 (3.3 ± 0.92), 18.7% on Day 70 (3.01 ± 0.88), and 19.0% on Day 90 (2.99 ± 1.1), as presented in Figure 3e. Student's *t* test (paired data) was used to calculate statistical significance of chromametry assessment. Improvements in L* and ITA° parameters along with reduction in ΔE* suggest the efficacy of the regimen in terms of skin brightening and even skin tone (skin homogeneity), respectively. PP analysis of chromametry demonstrated similar results and is presented in Figure S1c–e.

##### 3.2.2.5 CLBT Methodology

A significant increase (*p* < 0.001) in pink color saturation from baseline was recorded on Days 42, 56, 70, and 90, and a significant decrease (*p* < 0.001) in olive and yellow color saturations was recorded on Days 56, 70, and 90. In addition, posttreatment skin luminosity was significantly (*p* < 0.001) improved throughout the study; and improved brightness was evident, starting from Day 42 up to Day 90. Assessment of skin transparency demonstrated significant improvement (0.2%, *p* < 0.001) from baseline only at Day 90 (Table 2). Result of CLBT in PP analysis set is given in Table S1. Statistical significance was calculated using Student's *t* test (paired data).

##### 3.2.2.6 Subject's Self‐Evaluation Scores of the Regimen

**FIGURE 4:**
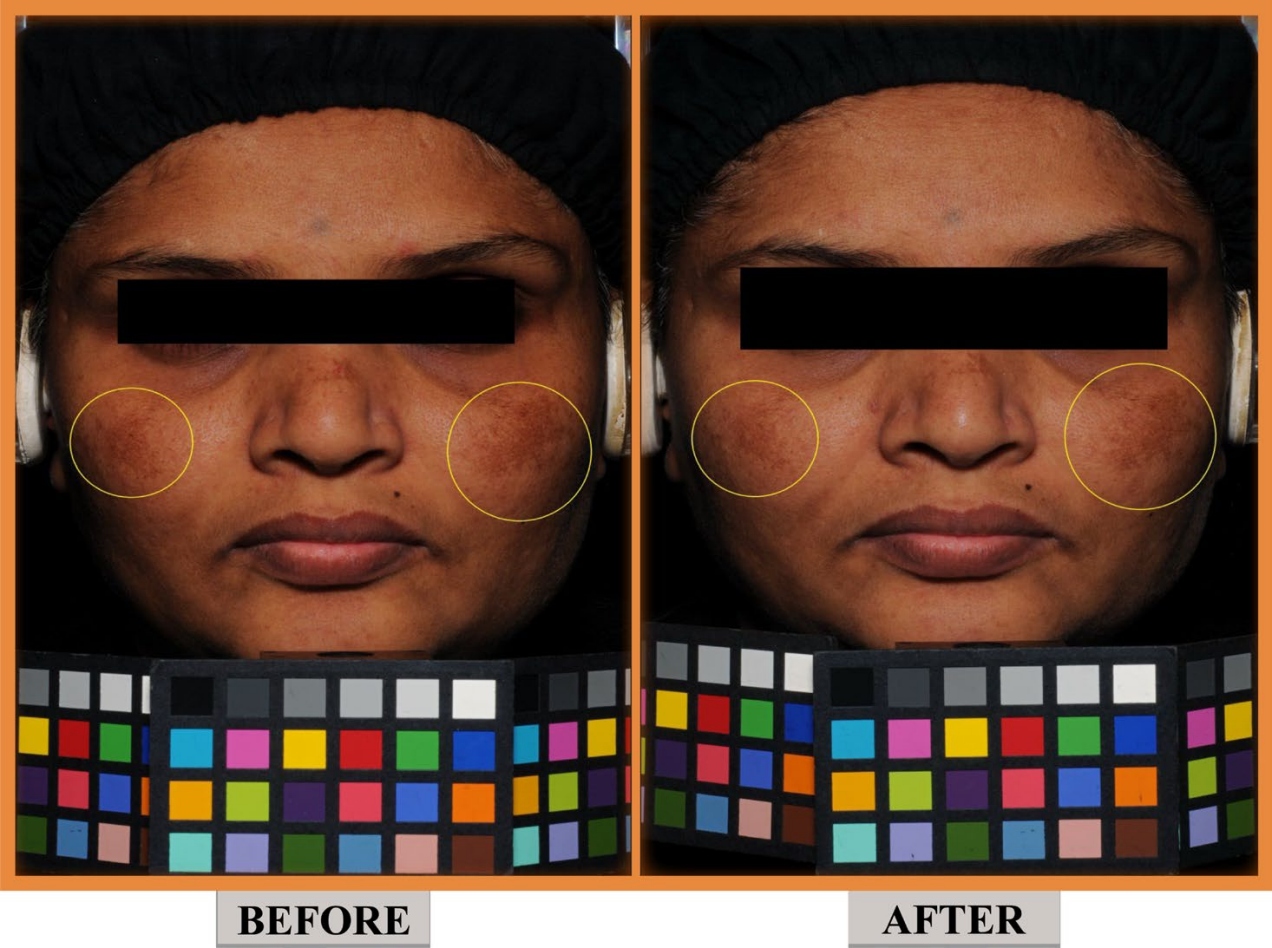
Representative images of before (left) and 90 days after (right) applying skincare regimen.

Subject's self‐evaluation scores for efficacy after using the skincare regimen are summarized in Table 3. The skincare regimen was significantly validated by subjects for making the skin bright (100%), even toned (100%), radiant (100%), and reducing pigmentary spots (dark spots [100%] or melasma [99%]). A representative image of subject's face before and 90 days after applying the skincare regimen is provided in Figure 4. Application of skincare regimen was significantly validated for absence of itching, irritation, and burning sensation on the skin with 100% agreement throughout the study (Table 3). Subject's self‐evaluation scores in PP analysis set are presented in Table S2.

**TABLE 3:** Panel agreement for product efficacy and safety based on subjects' self‐evaluation questionnaire (mITT analysis set).

Questionnaire	Panel agreement (%)				
	Day 28 (*n* = 119)	Day 42 (*n* = 117)	Day 56 (*n* = 112)	Day 70 (*n* = 113)	Day 90 (*n* = 113)
Efficacy evaluation					
The test product helps to brighten the skin	97	100	100	99	100
The test product helps to make the skin tone even	96	100	100	100	100
The test product helps to make the skin radiant	93	100	99	100	100
The test product helps to reduce the dark spots	93	100	100	100	100
The test product helps to reduce the melasma	91	100	100	100	99
Safety evaluation					
The test product does not cause itching on the skin	100	100	100	100	100
The test product does not cause irritation on the skin	100	100	100	100	100
The test product does not give burning sensation on the skin	100	100	100	100	100

### 3.3 Cosmetic Appeal of the Sunscreen Gel and Skincare Regimen

Cosmetic appeal questionnaire revealed that application of both sunscreen gel (at baseline) and skincare regimen (on Day 90) was well appreciated by 100% of the subjects (Table 4). Cosmetic appeal questionnaire in PP analysis set is given in Table S3.

**TABLE 4:** Self‐evaluation questionnaire for cosmetic appeal of sunscreen gel (*n* = 120) and skincare regimen (*n* = 113) by the study participants (mITT analysis set).

Cosmetic appeal questionnaire	Agreement (%)
Sunscreen gel (at baseline)	
The test product is nonsticky (no sticky residue on skin surface) on application	100
The test product is nonoily (without shininess) on application	100
The test product feels smooth (velvety) on application	100
The test product does not look white (white residue) on application	100
The test product gives matte finish (not shiny) on application	99
The test product feels light (weightless) on application	100
Skincare regimen (on Day 90)	
The fragrance of the test product is appealing	100
The test product quickly absorbs into the skin	100
The test product does not leave the skin sticky	100
The test product spreads properly on the face	100

#### 3.3.1 Safety

Dermatological evaluation for safety findings of skincare regimen demonstrated no unfavorable aggravation of clinical signs like, erythema, edema, dryness, scaling, peeling, itching, and tingling during the study. No adverse event was reported in the study.

## 4 Discussion

Awareness about photodamage and requirement of skin photoprotective measures is low among people with skin of color [21]. In such individuals, the more prevalent pigmentary disorder is melasma, that is, bilateral brown macules or patches that occur at sun‐exposed areas and are more common in females with skin types III and IV [22]. This negatively impacts the patient's emotional and psychological well‐being [23]. Clinical management of hyperpigmentation like dark spots and melasma is challenging for dermatologists, due to risk of safety side effects [10]. Retinoids, azelaic acid, hydroquinone, chemical peels, or cosmeceuticals, and prevention from sun exposure through the use of sunscreen and physical barriers are recommended for melasma treatment [10]. Hydroquinone and derivative of hydroquinone, that is, arbutin, are topically used due to their de‐pigmenting activity; arbutin has much lesser melanotoxic effects and high inhibitory actions on melanosome maturation [24]. Mathpati et al. [15] established the efficacy and safety of THBG and α‐arbutin containing formulation (Bristaa Intense) along with a sunscreen in Indian females. However, the study had a small sample size and short duration. Therefore, efficacy and safety of Bristaa Intense Cream and Photostable Gold Sunscreen Gel have been evaluated in a larger sample size in the present study.

Melanin content determines the human skin color. Noninvasive and in vivo measurement of the former is necessary to identify skin depigmentation and repigmentation [25]. Therapeutic efficacy and posttreatment improvement in clinical appearance of the patient are generally assessed based on melasma severity, using various well‐recognized objective tools [26]. As melanin absorbs a greater range of wavelengths including green, red, and near‐infrared light, degree of skin pigmentation is evaluated using mexameter that measures the difference in absorption/reflection between melanin and hemoglobin at specific wavelengths [27]. On 56th day of application of the skincare regimen, Mathpati et al. [15] found 11.0% and 46.1% reduction in melanin content and mMASI score of the pigmentary spots, respectively [15]. The current study showed significant decrease in the melanin content on mexametry readings by 11.9%, 15.3%, and 16.3% on 56, 70, and 90 days, respectively, indicating efficacy of the treatment regime in reducing the melanin content of the pigmentary spots. Moreover, the mMASI score started to improve significantly from Day 42 posttreatment (−5.1%) and was highest (−18.4%) on Day 90 after application of the regime. The lesser improvement in mMASI score in this study versus Mathpati et al. [15] can be attributed to higher baseline mMASI score in this study (4.8 vs. 3.34) [15]. The mean baseline mMASI score of 4.8 is consistent with findings of Arora et al. [28], who recorded a mean MASI score of 4.7 in Indian patients with melasma. The mean mMASI score in our study was recorded as 3.92 at the final follow‐up, indicating a subsequent reduction in area of melasma with the topical formulation and sunscreen.

Cross‐polarized light travels through the skin, scatters through multiple subsurfaces, depolarizes, and reflects to the camera [29]. This is especially efficacious for skin with high density of melanin, as the technique enhances visualization of the lesion in contrast to the underlying skin [30]. In the previous study, the total area of the pigmentary spot was decreased by 21.1% at Day 56; no significant change in ΔE*ab was observed [15]. Cross‐polarized photographs of patients in this study demonstrated no change in morphology parameters (number of pigmentary spots and total area of pigmentary spots). However, a significant decrease in the ΔEab parameter depicted improvement in color difference between pigmentary spot and normal skin by 14.3%, 12.3%, and 8.7% at 56, 70, and 90 days, respectively. In addition, the results of chromametry showed an improvement in skin brightness, indicated by a significant increase in L* (0.9%, 1.4%, 2.0% 2.2%, and 2.7% after 28, 42, 56, 70, and 90 days, respectively) and ITA° (12.7%, 18.8%, 28.1%, 30.6%, and 37.7% after 28, 42, 56, 70, and 90 days, respectively) values. Effect of the treatment with skincare regimen in improvement of skin tone evenness or skin homogeneity was reflected by a significant decrease in ΔE value by 6.0%, 11.1%, 18.7%, and 19.0% on Days 42, 56, 70, and 90, respectively. Mathpati et al. [15] found significant skin‐brightening effect with the skincare regimen as 1.12% and 15.4% increase in L* and ITA° by 56th day was recorded [15]. Similar to this study, Draelos et al. [31] reported improvement in skin tone evenness by 19% at Week 8 after applying a topical facial formulation (containing hexylresorcinol, silymarin, and vitamin C and E) in UVB‐exposed skin explants.

The present results suggest a positive effect of the topical formulation along with sunscreen on skin radiance. Skin color, skin luminosity, brightness, and transparency were improved and pigmentation was reduced. A significant increase in pink color saturation by 32.8%, skin luminosity by 6.0%, skin brightness by 4.6%, and skin transparency by 0.2%, and a significant decrease in yellow and olive color saturations by 5.5% and 7.8%, respectively, after 90 days of the skincare regimen application resulted in improved skin radiance. Furthermore, efficacy, safety, and cosmetic appeal of the products were also derived from subject's self‐evaluation questionnaire or panel agreement on the products. Subject self‐evaluation score indicated that all subjects validated the treatment regime with both products in reduction of melasma or dark spots, and improvement in skin brightening, tone, and radiance at all visits. Safety analysis also depicted 100% agreement on no itching, irritation, and burning sensation on the skin associated with the product use, and no unfavorable aggravation of erythema, edema, dryness, scaling, peeling, itching, and tingling on dermatological evaluation at any time point during the study. Unlike this study, the study by Mathpati et al. [15] noted mild dryness in one subject on Day 28 and product‐related redness on the cheeks and forehead on Day 56 [15]. However, study participants of the current study had well appreciated the sunscreen gel as nonsticky, nonoily, nonshiny, velvety, weightless, and for not leaving white residue on the skin. Subjects' self‐evaluation at final follow‐up for cosmetic appeal showed subjects' favorable opinion for fragrance, quick absorption, nonsticky, and proper spreadability of the product. In addition, no incidence of adverse or serious adverse events confirmed tolerability and safety of the study products.

A noncomparative nature of the study was its limitation. However, use of objective quantitative and qualitative tools like mexameter, mMASI, chromameter, and cross‐polarized light photography for product efficacy measurement is the key strength of this study. The additional strength is the longer duration of study. A randomized, comparative, double‐blind study in larger sample size may be needed to confirm these findings.

Application of skincare regimen (containing 10% THBG and 2% α‐arbutin along with Photostable Gold Sunscreen Gel) for 90 days reduced melanin content (on mexametry) and visibility of pigmentary spots (dark spots or melasma), and improved mMASI score, skin brightness, skin tone, and radiance. Dermatologist's complete agreement on clinical and functional signs related to the product, and no recorded incidence of adverse events suggested tolerability and safety of skincare regimen.

The results of this study demonstrated that the skincare regimen including 10% THBG and 2% arbutin (Bristaa Intense) along with sunscreen (Photostable Gold Gel) was effective in reducing pigmentary spots (dark spots and melasma) and was well tolerated in Indian women.

## Author Contributions

R.P. conducted the study. R.P. was involved in acquisition, analysis, and interpretation of data, and drafting of manuscript. M.G., P.S., C.K., and S.M. were involved in conceptualization, designing the study, analysis, and interpretation of data. S.D. was involved in interpretation of data and drafting and revising the manuscript. All authors reviewed and approved the final version of manuscript.

## Ethics Statement

The study was registered in the Clinical Trial Registry of India (CTRI Reg. No. CTRI/2021/11/038345; date: 30/11/2021), and conducted as per ICH GCP and all the applicable regulatory requirements. The protocol and study documents were reviewed and approved by the Institutional Ethics Committee of the participating site, Mascot Spincontrol, named “ETHOS.” The Ethics committee also monitored the overall progress of the study. Consolidated Standards of Reporting Trial (CONSORT) guidelines were also followed.

## Consent

A written permission was obtained for publishing clinical pictures by the patients.

## Conflicts of Interest

The authors declare no conflicts of interest. Author R.P. is a full time employee of Mascot Spincontrol India Pvt. Ltd., Mumbai, India, where the study was conducted.

## Supporting information


Figure S1.

Appendix S1.

## Data Availability

The data that support the findings of this study are available on request from the corresponding author. The data are not publicly available due to privacy or ethical restrictions.
