# Supplementary figures and images for: Efficacy and Safety of a Topical Formulation Containing Trihydroxybenzoic Acid Glucoside and α‐Arbutin, Applied Along With a Sunscreen: A Noncomparative, Prospective, Interventional Study in Indian Females With Facial Melasma or Dark Spots
Source: J Cosmet Dermatol. 2025 Feb 12;24(2):e70017. doi: 10.1111/jocd.70017 (PMC11822242; doi:10.1111/jocd.70017)

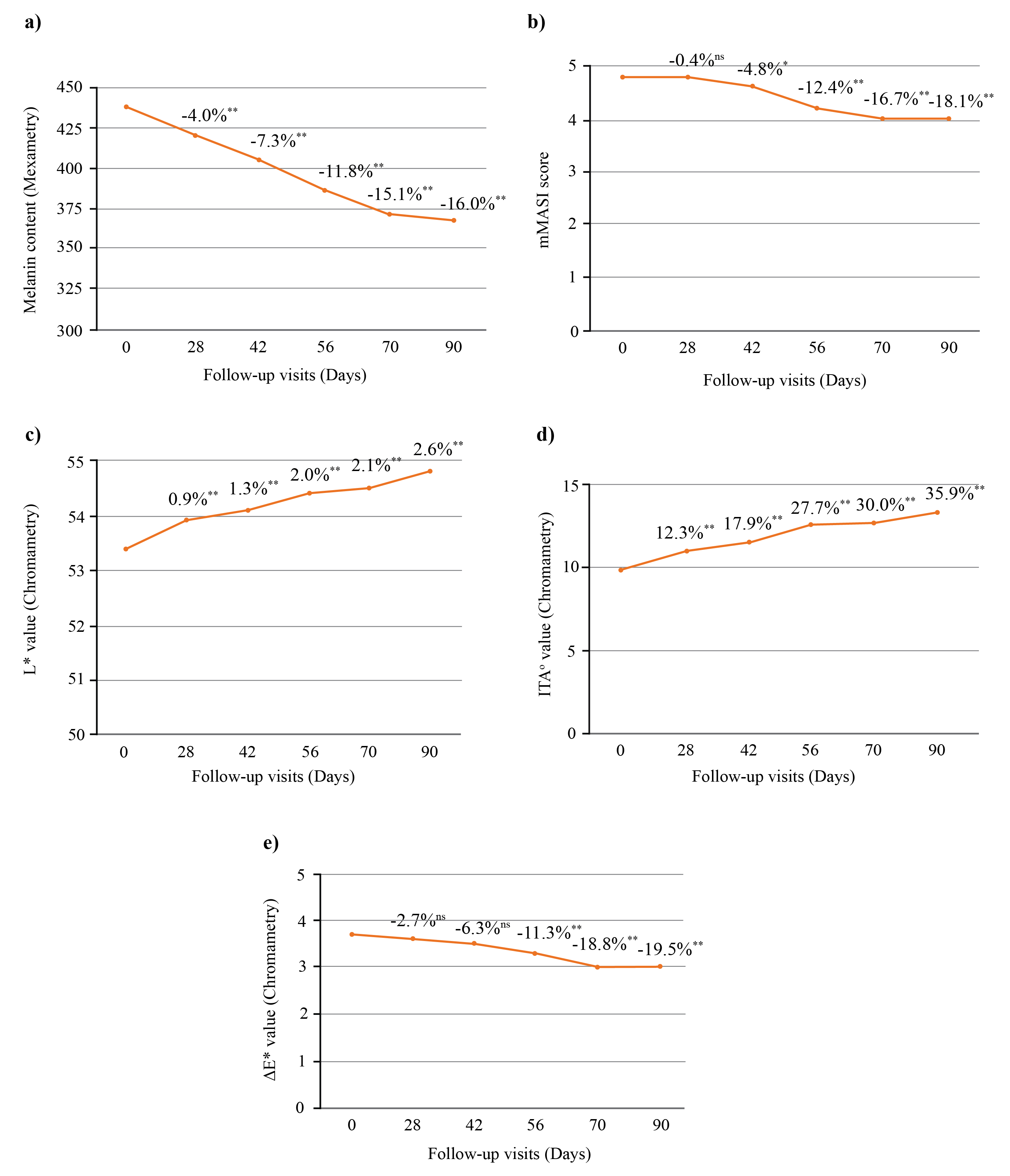

Supplement: Supplementary file 1 — Figure S1. [file JOCD-24-e70017-s001.png]
