# Supplementary material for: Efficacy and Safety of a Topical Formulation Containing Trihydroxybenzoic Acid Glucoside and α‐Arbutin, Applied Along With a Sunscreen: A Noncomparative, Prospective, Interventional Study in Indian Females With Facial Melasma or Dark Spots
Source: J Cosmet Dermatol. 2025 Feb 12;24(2):e70017. doi: 10.1111/jocd.70017 (PMC11822242; doi:10.1111/jocd.70017)
Supplement: Supplementary file 2 — Appendix S1. [file JOCD-24-e70017-s002.docx]

Supplementary Tables

Supplementary Table 1: Evaluation of efficacy parameters in the study participants (n=109) (PP analysis set)

| **Endpoints** | **Study visit** | **Mean±SD** | **Average change vs. baseline** | **p value vs. baseline** |
| --- | --- | --- | --- | --- |
| ***Cross polarized light photography*** | | | | |
| ΔEab | Day 0 | 8.6±1.97 |  |  |
|  | Day 56 | 7.37±1.86 | -14.2% | <0.001** |
|  | Day 70 | 7.51±1.85 | -12.6% | <0.001** |
|  | Day 90 | 7.83±1.92 | -8.9% | <0.001** |
| Number of spots in region of interest | Day 0 | 2.56±2.11 |  |  |
|  | Day 56 | 2.76±2.55 | 7.9% | 0.26 |
|  | Day 70 | 2.4±2.33 | -6.1% | 0.28 |
|  | Day 90 | 2.51±1.96 | -1.8% | 0.80 |
| Total area occupied by spots (pixels^2^) | Day 0 | 14174.74±15523.16 |  |  |
|  | Day 56 | 14346.18±15111.31 | 1.2% | 0.54 |
|  | Day 70 | 13960.28±14477.52 | -1.5% | 0.62 |
|  | Day 90 | 14671.92±16359.33 | 3.5% | 0.29 |
| ***Skin radiance by CLBT methodology*** | | | | |
| Pink color | Day 0 | 10.93±7.01 |  |  |
|  | Day 28 | 11.01±7.13 | 0.70% | 0.5 |
|  | Day 42 | 11.65±7.66 | 6.57% | <0.001** |
|  | Day 56 | 12.83±7.77 | 17.34% | <0.001** |
|  | Day 70 | 13.75±8.17 | 25.73% | <0.001** |
|  | Day 90 | 14.42±8.44 | 31.89% | <0.001** |
| Yellow color | Day 0 | 35.46±5.46 |  |  |
|  | Day 28 | 35.31±5.48 | -0.43% | 0.5 |
|  | Day 42 | 35.49±5.15 | 0.09% | 1.0 |
|  | Day 56 | 34.33±4.62 | -3.19% | <0.001** |
|  | Day 70 | 33.72±4.17 | -4.92% | <0.001** |
|  | Day 90 | 33.44±4.07 | -5.69% | <0.001** |
| Olive color | Day 0 | 33.69±4.18 |  |  |
|  | Day 28 | 33.70±4.18 | 0.05% | 1.0 |
|  | Day 42 | 33.62±4.09 | -0.18% | 0.38 |
|  | Day 56 | 32.13±3.56 | -4.63% | <0.001** |
|  | Day 70 | 31.48±3.50 | -6.54% | <0.001** |
|  | Day 90 | 31.04±3.53 | -7.85% | <0.001** |
| Luminosity | Day 0 | 3.79±0.22 |  |  |
|  | Day 28 | 3.81±0.23 | 0.47% | <0.001** |
|  | Day 42 | 3.90±0.24 | 2.73% | <0.001** |
|  | Day 56 | 3.96±0.25 | 4.29% | <0.001** |
|  | Day 70 | 3.99±0.25 | 5.16% | <0.001** |
|  | Day 90 | 4.02±0.26 | 5.99% | <0.001** |
| Brightness | Day 0 | 3.73±0.37 |  |  |
|  | Day 28 | 3.73±0.37 | 0.01% | 1.0 |
|  | Day 42 | 3.78±0.38 | 1.35% | <0.001** |
|  | Day 56 | 3.85±0.38 | 3.29% | <0.001** |
|  | Day 70 | 3.88±0.38 | 3.99% | <0.001** |
|  | Day 90 | 3.90±0.38 | 4.63% | <0.001** |
| Transparency | Day 0 | 3.62±0.31 |  |  |
|  | Day 28 | 3.62±0.31 | 0 | 1.0 |
|  | Day 42 | 3.62±0.31 | 0.05% | 0.13 |
|  | Day 56 | 3.62±0.31 | 0.05% | 0.31 |
|  | Day 70 | 3.63±0.31 | 0.10% | 0.06 |
|  | Day 90 | 3.63±0.31 | 0.18% | <0.001** |

Student t test (paired data) used for calculation of p value. **p<0.001

Supplementary Table 2: Panel agreement for product efficacy and safety based on subjects’ self evaluation questionnaire (n=109) (PP analysis set)

| **Questionnaire** | **Panel agreement (%)** | | | | |
| --- | --- | --- | --- | --- | --- |
|  | **Day 28** | **Day 42** | **Day 56** | **Day 70** | **Day 90** |
| ***Efficacy evaluation*** |  |  |  |  |  |
| The test product helps to brighten the skin | 97 | 100 | 100 | 99 | 100 |
| The test product helps to make the skin tone even | 95 | 100 | 100 | 100 | 100 |
| The test product helps to make the skin radiant | 93 | 100 | 99 | 100 | 100 |
| The test product helps to reduce the dark spots | 93 | 100 | 100 | 100 | 100 |
| The test product helps to reduce the melasma | 90 | 100 | 100 | 100 | 99 |
| ***Safety evaluation*** |  |  |  |  |  |
| The test product does not cause itching on the skin | 100 | 100 | 100 | 100 | 100 |
| The test product does not cause irritation on the skin | 100 | 100 | 100 | 100 | 100 |
| The test product does not give burning sensation on the skin | 100 | 100 | 100 | 100 | 100 |

Supplementary Table 3: Self-evaluation questionnaire for cosmetic appeal of sunscreen gel and skincare regimen by the study participants (n=109) (PP analysis set)

| **Cosmetic appeal questionnaire** | **Agreement (%)** |
| --- | --- |
| ***Sunscreen gel (at baseline)*** | |
| The test product is non-sticky (no sticky residue on skin surface) on application | 100 |
| The test product is non-oily (without shininess) on application | 100 |
| The test product feels smooth (velvety) on application | 100 |
| The test product does not look white (white residue) on application | 100 |
| The test product gives matte finish (not shiny) on application | 99 |
| The test product feels light (weightless) on application | 100 |
| ***Skincare regimen (on Day 90)*** | |
| The fragrance of the test product is appealing | 100 |
| The test product quickly absorbs into the skin | 100 |
| The test product does not leave the skin sticky | 100 |
| The test product spreads properly on the face | 100 |

# SUPPLEMENTRY FIGURE LEGEND

Supplementry Figure 1: Percent change from baseline (Day 0) in (a) melanin content through mexametry, (b) mMASI score, (c) L* value, (d) ITA° value and (e) ΔE* value in the study participants (n=109). Student t test (paired data) was used to calculate statistical significance. ^ns^Not significant, *p<0.01, **p<0.001
